# An Optimized GD2-Targeting Retroviral Cassette for More Potent and Safer Cellular Therapy of Neuroblastoma and Other Cancers

**DOI:** 10.1371/journal.pone.0152196

**Published:** 2016-03-31

**Authors:** Simon Thomas, Karin Straathof, Nourredine Himoudi, John Anderson, Martin Pule

**Affiliations:** 1 Cancer Institute, University College London, London, United Kingdom; 2 Institute of Child Health, University College London, London, United Kingdom; King's College London, UNITED KINGDOM

## Abstract

Neuroblastoma is the commonest extra cranial solid cancer of childhood. Despite escalation of treatment regimens, a significant minority of patients die of their disease. Disialoganglioside (GD2) is consistently expressed at high-levels in neuroblastoma tumors, which have been targeted with some success using therapeutic monoclonal antibodies. GD2 is also expressed in a range of other cancer but with the exception of some peripheral nerves is largely absent from non-transformed tissues. Chimeric Antigen Receptors (CARs) are artificial type I proteins which graft the specificity of a monoclonal antibody onto a T-cell. Clinical data with early CAR designs directed against GD2 have shown some promise in Neuroblastoma. Here, we describe a GD2-targeting CAR retroviral cassette, which has been optimized for CAR T-cell persistence, efficacy and safety.

## Introduction

Neuroblastoma accounts for approximately 15% of cancer deaths in children [1]. Despite marked intensification of therapy, less than 40% of high-risk patients are long-term survivors, with chemotherapy and radiotherapy resistance and late relapses being the hallmark of treatment failure [2]. Disialoganglioside (GD2), a surface glycolipid antigen that is ubiquitous and abundant on neuroblastoma cells, as well as having cancer-specific expression in a number of adult and paediatric malignancies [3], is an ideal target for immunotherapy [4]. Indeed, anti-GD2 monoclonal antibodies currently form part of standard treatment for high risk neuroblastoma, and their efficacy and toxicity profile is well-established [3,5].

Administration of tumor-specific T-cells (adoptive immunotherapy) has proven to be an effective cancer treatment for Epstein Barr virus-driven lymphomas [6] and melanoma [7] with responses in bulky resistant disease. However, it has not been possible to generate neuroblastoma specific T-cells using traditional methods of selection and expansion. Chimeric Antigen Receptors (CARs) can be constructed by connecting the single-chain variable region (scFv) from a monoclonal antibody to intracellular signalling domains. GD2-targeting CARs therefore afford us an alternative method of generating neuroblastoma specific T-cells by genetic engineering. GD2 CAR therapy may result in improved responses over mAb therapy due to a persisting and dynamic rejection of GD2-expressing tumor.

A phase I clinical study of anti-GD2 CAR transduced T-cells in relapsed high risk neuroblastoma patients reported some efficacy [8]. A possible limitation of that study was the use of a first generation CAR, providing only CD3ζ ITAM signals, which may have resulted in poor persistence and expansion. An increasing body of clinical data of CD19 CAR in B-cell malignancies as well as a double-marking study [9] suggest that CARs providing additional co-stimulatory signals result in improved persistence and efficacy. Here, we describe our efforts to construct a more potent but safe GD2-targeting cassette for use against neuroblastoma, which utilizes a previously described third generation endodomain [10]. The focus of this work is optimization of the remaining CAR architecture and expression cassette for maximal efficacy and safety.

The CAR investigated in the study reported by Pule et al used an scFv derived from 14–18, a mAb which in a chimeric form is currently in regular clinical use. We have therefore used a targeting domain from a different anti-GD2 mAb family to avoid anti-idiotype rejection/activation of CAR T-cells. To reduce the chance of rejection, a humanized version of the CAR was tested, and iterative optimization of the CAR architecture was performed. Anti-GD2 mAb therapy is associated with peripheral neurotoxicity. While the initial GD2 CAR study did not report this [8], the concern lingers as increasingly potent CARs are introduced into the clinic. In anticipation of this eventuality, we co-expressed CAR with the iCasp9 suicide gene [11] and optimized a bi-cistronic retroviral cassette to maintain co-expression and consistent transgene output. The final construct was tested in vivo. We have generated a GD2 CAR targeting retroviral cassette optimized for efficacy and safety.

## Results

### CAR with humanized scFv gives similar expression and increased cytokine release and T-cell expansion

**Fig 1:**
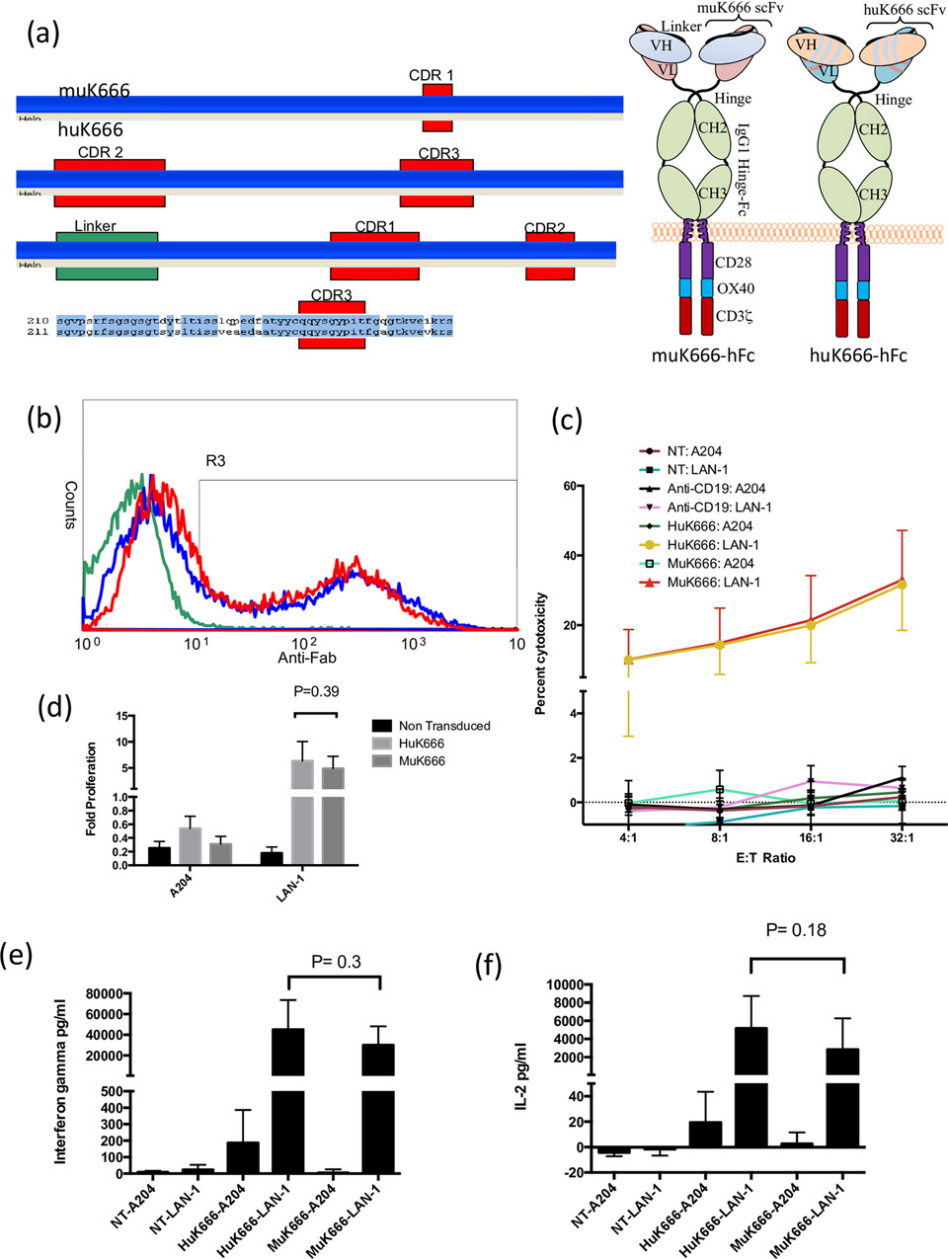
Comparison between huK666 and muK666 based CARs. (a) Comparison of amino acid sequences of huK666 and muK666 scFvs are compared. Complementarity determining regions are shown in red. The linker sequence is shown in green. On right, architectures of the CARs generated for initial comparison of huK666 with muK666. Both differ only in the scFvs used being either the original murine muK666 sequences, or the humanized (CDR grafted) huK666. Otherwise the CARs comprise of a human IgG1 hinge-CH2-CH3 spacer, a TM domain derived from CD28 and a compound endodomain comprising of fusions between endodomains from CD28, OX40 and CD3-Zeta. (b) Stability of muK666 vs huK666 CARs. T-cells from 5 donors were transduced with equal titers of retroviral supernatant coding for muK666 or huK666 CARs. CAR expression was detected by anti-human-Fc polyclonal antibodies. MFI was identical in both constructs. A representative example is shown with NT (green), muK666 (blue) and huK666 (red) histograms overlaid. These CAR T-cells were challenged with LAN-1 (a Neuroblastoma cell line expressing GD2) and A204 (a rhabdomyosarcoma cell line which is GD2 negative). Cytotoxicity is shown in (c), proliferation at day 7 is shown in (d), IFN-γ in (e) and IL-2 release in (f). Data shown as means +/-SEM from 6 independent experiments with different donors.

KM8138 is a fully humanized anti-GD2 monoclonal antibody constructed by grafting the epitope binding complementarity determining regions (CDRs) of the murine anti-GD2 antibody KM666 onto compatible human V_H_ and V_L_ framework regions [12]. The resultant human scFv sequence differs from the murine in 31 residues in the framework regions outside of the CDRs. Murine antibody 14.18-derived scFv used in previously described GD2 CARs may be a target for immune rejection either due to anti-idiotype (since therapeutic mAbs in current clinical use are derived from the same clone), or from anti-mouse antibodies. We therefore derived a CAR based on the humanized KM8138 antibody [12]. To determine any consequences of using a humanized scFv, we also generated a CAR derived from the parental mouse antibody thus we generated a pair of anti-GD2 CARs identical except for their scFvs, which were derived from a murine anti-GD2 mAb KM666, or its humanized counterpart KM8138 [12]. The CARs had human IgG1 hinge-Fc spacers, the CD28 transmembrane domain and the CD28-OX40-Zeta endodomain (Fig 1A). Initially we sought to compare the expression and function of this humanized CAR (HuK666) with its murine counterpart (MuK666). Normal donor human T-cells were transduced with equal titers of retroviral vector coding for each receptor. Surface expression of each CAR determined by flow-cytometry with a polyclonal anti-Fc was identical (Fig 1B). Mean fluorescence intensity (MFI) of transduced T-cells did not differ between humanized and murine CARs. Transduced T-cells were In a 4-hour chromium release assay CD56-depleted T-cells expressing either CAR demonstrated comparable killing of the GD2-bearing Lan-1 neuroblastoma cell line and an equivalent absence of activity against A204 osteosarcoma cell line which lacks expression of GD2. Neither non-transduced T cells (NT) nor T cells transduced with an irrelevant CAR directed against CD19 displayed any cytotoxicity to either cell line (Fig 1C) Both receptors were capable of stimulating the proliferation of transduced T cells as wells as the secretion of IL2 and IFN-g in response to Lan1 but not A204 (Fig 1D). There was no significant difference between huK666 and muK666 CARs for proliferation (Fig 1D), interferon γ release (Fig 1E), or interleukin 2 (IL-2) release (Fig 1F) although there was a non significant trend for all three towards enhanced function of the huK666 CAR.

### Spacer comprising of IgG1 Fc domain results in optimal killing and cytokine release

**Fig 2:**
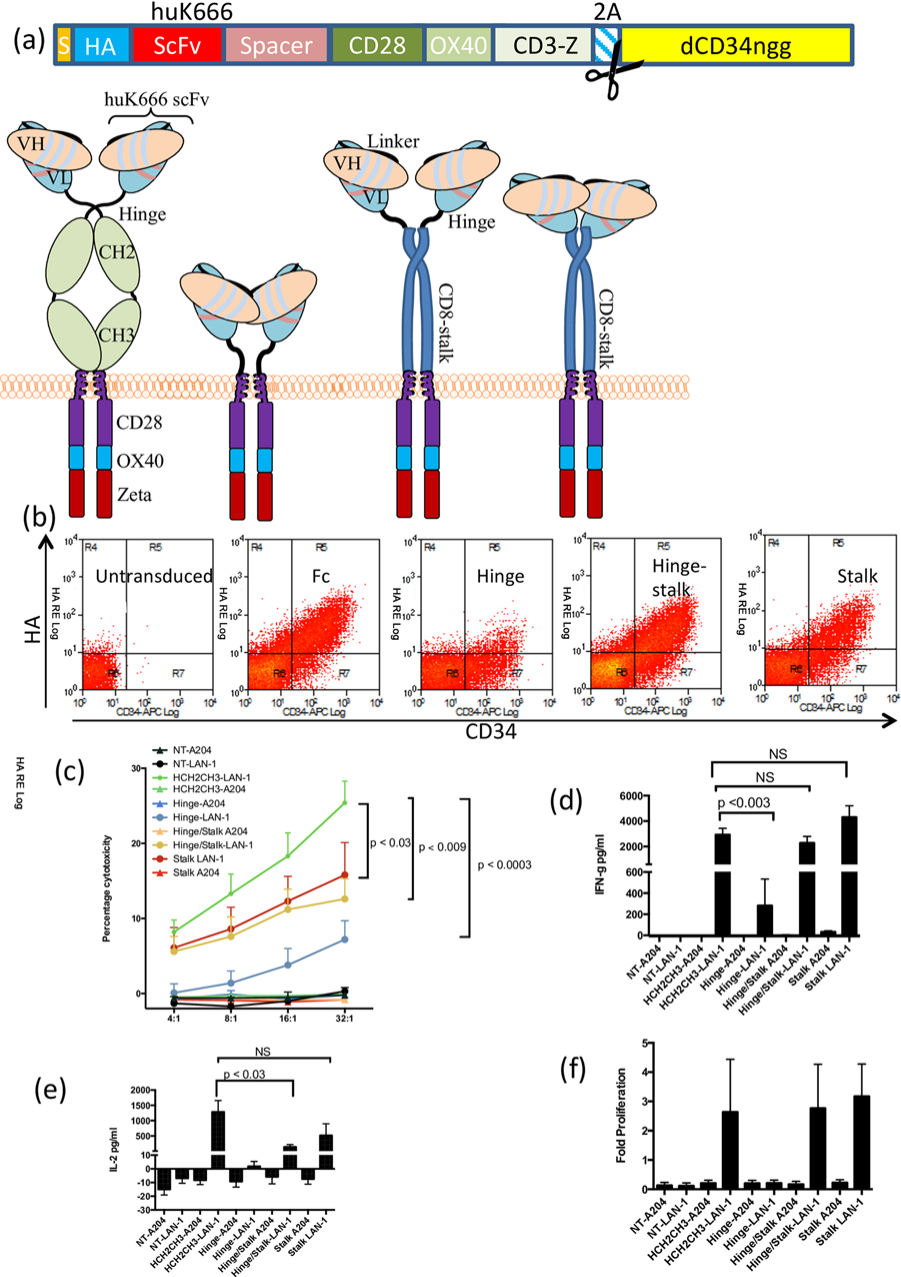
Effects of spacer on CAR function. Several huK666 CARs were cloned into an identical format whereby the scFv was tagged with an HA-tag, and the CAR was co-expressed with a truncated FMD-2A sequence with truncated CD34. The expression cassette is shown in (a) and cartoons of these formats are shown in (b). Co-expression of CAR (HA) and CD34 marker gene from the different cassettes. The four hinge regions were compared in terms of cytotoxicity (c) (Lan1 = GD2 positive target and A204 = GD2 negative target), IFN-γ secretion (d), IL-2 (e) and target-specific proliferation (f). Data shown as means +/-SEM from 4 independent experiments with different donors.

CAR function can be influenced by spacer domain selection [13–15]. In addition to the huK666-based CAR described above containing the human IgG1 hinge-CH2-CH3 domain as spacer, we generated additional huK666 CAR variants in which the spacer region was comprised of the hinge alone, the hinge attached to the stalk of CD8a or the CD8a stalk alone (Fig 2A). Selection of these spacers was based on size consideration, with IgG1 hinge-alone being a short spacer, CD8 stalk intermediate and IgG1 Fc being a long spacer. We also postulated that the CD8 stalk may not allow sufficient articulation of the scFv and hence we generated an additional variant with the IgG1 hinge connected to the CD8 stalk. To allow easy detection of CAR, we tagged the huK666 with an amino-terminal HA-tag, inserted between the signal-peptide and the VH. Further, to control for variation in vector expression, we cloned the foot-and-mouth disease 2A sequence TaV (2ATaV) in frame with the carboxy-terminus of the CAR, which in turn was cloned in frame to truncated CD34 (dCD34ngg). We could therefore detect CAR expression relative to vector expression by staining for HA and anti-CD34.

Primary human T-cells were transduced with equal titer supernatant from these constructs and stained with anti-HA and anti-CD34. A representative flow-cytometric analysis of this staining is shown in Fig 2B. Hinge-CH2-CH3, hinge-Stalk and Stalk spacer CARs expression were identical, while the hinge-alone spacer CAR resulted in reduced HA staining (Fig 2B). This might be due to reduced stability, or to reduced access to the HA-tag in this format. Functional experiments were performed next, and there were clear differences in the abilities of the spacer variants to mediate cytotoxicity, cytokine release and proliferation in response to Lan-1 cells. PBMCs transduced with any of the spacer variant CARs displayed cytotoxicity toward Lan-1 cells; however there were significant differences between efficacy depending on spacer. CAR expressing the CH2-CH3 spacer were significantly more effective than hinge/stalk (P = 0.009) and hinge (p = 0.0003) although the trend to enhanced killing compared with stalk was non-significant. None of the spacer variant CARs mediated any killing of GD2 negative A204 cells (Fig 2C). Differences were also observed in the ability of the other spacer variants to stimulate the production of IFN-γ or IL-2 following culture with Lan1 cells. CH2CH3 spacer consistently induced both of the cytokines, and was significantly better than hinge for IFN-γ production (p<0.003, Fig 2D), and significantly better than hinge/stalk (p<0.03) and hinge (p<0.006) for IL-2 production (Fig 2E). With the exception of the hinge variant all receptors seemed comparable in terms of their ability to stimulate cell proliferation in response to GD2-bearing Lan1 despite substantial variation between donors (Fig 2F). T-cells expressing the hinge variant showed little detectable proliferation, which is consistent with the reduced ability of this receptor to mediate IL-2 release. Overall the IgG Fc region appeared to be the optimal spacer for the GD2 CAR and we used this receptor format in subsequent optimizations.

### Enhanced CAR function through modification of Spacer

**Fig 3:**
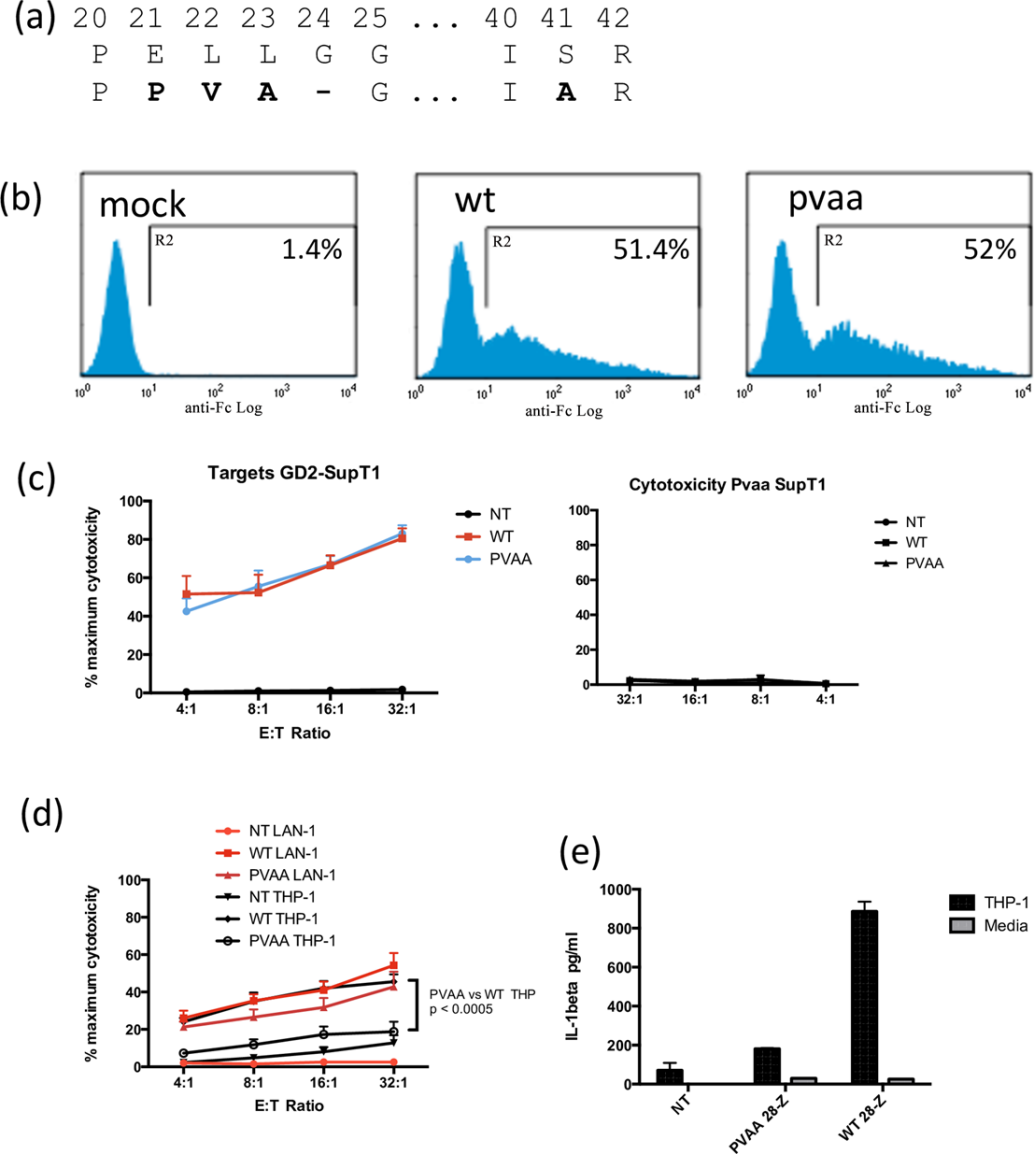
Removal of FcR binding motifs in IgG1 spacer. (a) amino acid sequence of the wild type (top) and mutated (PVAA) CH2 regions responsible for FcγR binding. (b) flow staining with anti-Fc antibody to show comparable level of expression of receptor with or without the PVAA mutation. Identical CARs with and without PVAA were compared side by side in terms of cytotoxicity against GD2 engineered SupT1 and GD2 negative wild type SipT1 cells (c), cytotoxicity against GD2 positive Lan1 cells and FcRγ positive THP-1 cells (d), and IL-1β release on culture with THP-1 cells (e). Data shown as means +/-SEM from 4 independent experiments with different donors.

A normal function of the CH2-CH3 region of the Fc region of immunoglobulins is the binding of Fc receptors on immune effector cells. The human IgG1 CH2-CH3 spacer used in the GD2 CAR is the natural ligand for high affinity FcγRI (CD64) expressed on innate effectors such as macrophages and monocytes. Hence there is a theoretical risk of engagement of CAR expressing T cells with myeloid cells by binding of CD64 resulting in both off target toxicity of myeloid cells, and diversion of the CAR T cells from their intended effector function. Indeed this phenomenon of CARs containing IgG1 CH2-CH3 spacers has previously been reported [16,17]. The critical amino acids for IgG1 recognition reside in CH2 domain (PELLGG and ISR motifs [18]), and Hombach et al have shown that replacement of these key amino acids with the corresponding amino acids from IgG2 within the context of CAR design, has no inhibitory effect in vitro of a CD30 targeting CAR, but prevents off target lysis of CD64-expressing THP1 cells [16]. Therefore we mutated the PELLGG and ISR residues as per the approach of Hombach et al (Fig 3A) and demonstrated equivalent expression in T cells of CAR carrying the mutated (abbreviated to PVAA) CAR (Fig 3B). The PVAA-mutated CAR retained antigen specific effector function against SupT1 cells engineered to express GD2 at bright levels (Fig 3C). Whilst the PVAA-containing CAR lysed GD2-expressing LAN1 cells with equivalent efficacy to wild type, the PVAA CAR had significantly reduced off target cytolysis against CD64 expressing THP1 cells (p = 0.0005 Fig 3D). Similarly, whilst co-culture of WT-CAR T cells with THP1 cells resulted in mean detectable IL1β in supernatant of 886pg/ml, this was decreased to values that were not above background with PVAA containing CAR (Fig 3E). Therefore the incorporation of a mutation to prevent binding of high affinity FcγR avoids off target toxicity by the GD2-CAR.

### Optimization of co-expression with suicide gene

**Fig 4:**
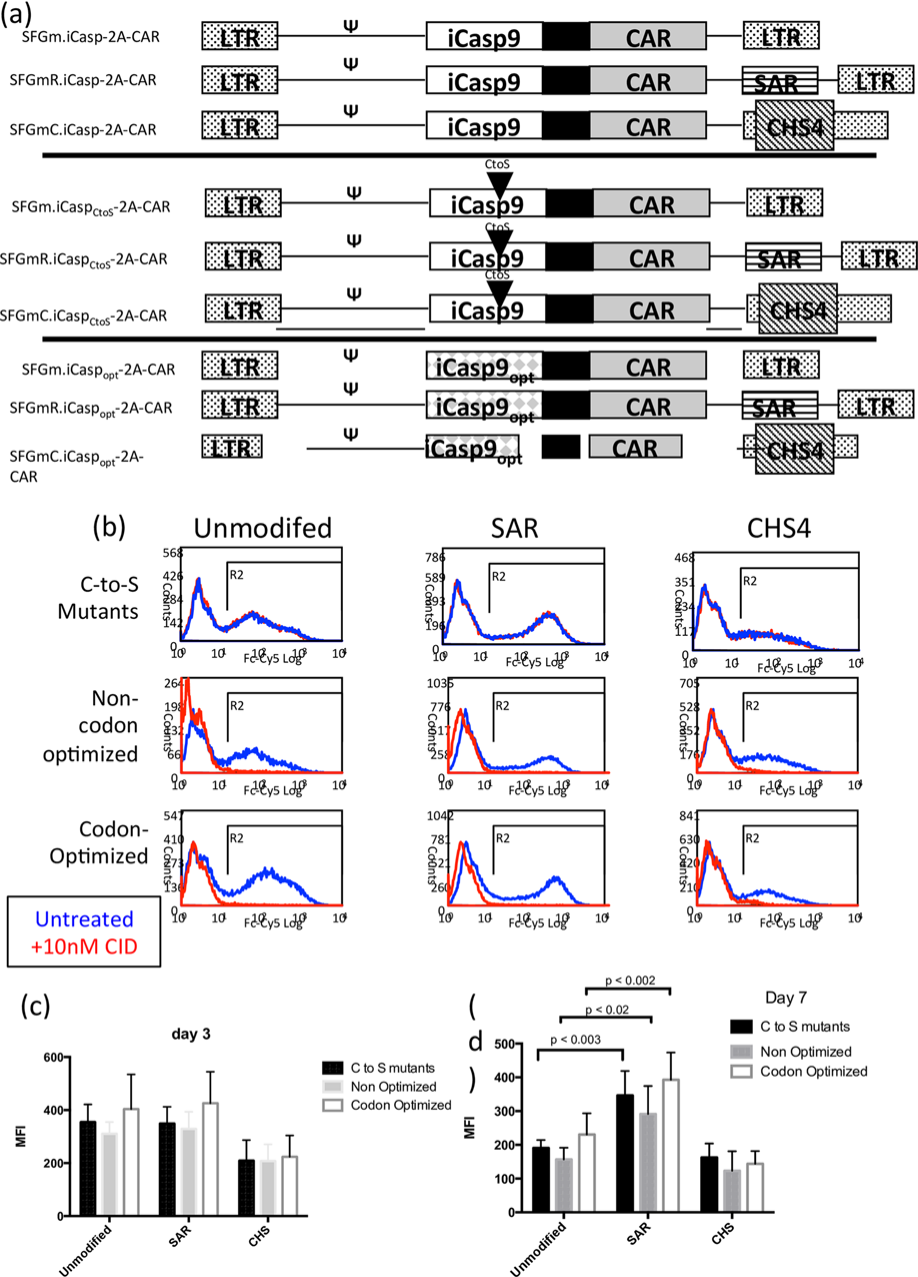
Optimization of expression cassette for co-expression with iCasp9. (a) Nine different expression cassettes were compared: Three different retroviral vectors were generated–wild-type SFG, SFG with scaffold-attachment region (SAR) inserted into the 3’UTR and SFG with CHS4 inserted into the 3’LTR U3 region. (retroviral vectors with both SAR and CHS4 were generated but produced very low vector titers and were not compared further); Into these retroviral vectors wild-type iCasp9-2A-huK666 CAR constructs were inserted and iCasp9 were inserted in 3 forms; codon-optimized, wild-type, and with the catalytic domain mutated. (b) Histogrammes at day 3 after transduction (blue lines). Overlaid T-cells cultured in 20nM CID are shown in red. Bargraphs of MFI of cells in the absence of CID at day 3 (c) and (d) day 7. Data shown as means +/-SEM from 5 independent experiments with different donors.

Although providing a powerful potential treatment for cancer, CAR-T cells have the capacity to mediate potentially fatal adverse events. GD2 targeting may lead to on-target off-tumour toxicity caused by central and peripheral nervous system GD2 expression. A means to selectively delete CAR T-cells in the face of unacceptable toxicity is desirable. To achieve this, we co-expressed the inducible Caspase 9 (iCasp9) suicide gene [11]. Co-expression of CAR and iCasp9 was using the self-cleaving FMD-2A like sequence TaV^13^. This results in obligate 1:1 co-expression of CAR with suicide gene. Since retroviral expression results in a normal distribution of expression intensity, one consideration is escape of low level iCasp9 expressing CAR T-cells. In addition, high levels of iCasp9 may be basally toxic. Cells expressing low-level iCasp9 can survive suicide gene activation, so we sought to achieve homogenous bright expression of CAR and iCasp9, despite the additional transcriptional burden that the 1.5 kb iCasp adds to the vector, and to show that iCasp9 was not basally toxic at achieved expression levels. We modified the vector and open reading frames as follows: Vector 3’LTR U3 was modified to include the chicken β-globin chromatin insulator [19]. The Scaffold attachment region from the human interferon-β gene was inserted into the 3’UTR [20] (Fig 4A). We also generated constructs with both modifications together but titres were too low to be useable and were not tested further (data not shown). Additionally, the entire open reading frame was codon-optimized. Finally, to ensure that iCasp9 did not result in basal toxicity at high-expression levels we generated a non-functional mutant with the active site cysteine mutated to serine (Fig 4A). PBMCs were transduced with equal titres of retroviral supernatant for each receptor, and expression was monitored 72 hour post-transduction by flow cytometry. The mean fluorescence intensity of each variant fused to the non-functional iCasp9 is slightly greater than the corresponding non-optimized receptor linked to a functional iCasp9, suggesting highly expressing cells may be lost due to inappropriate, non-specific activation of the iCasp9 (Fig 4B and 4C).

To evaluate the effects of vector modifications over more prolonged periods, transduced PBMCs were then cultured for a total of 7 days. Over the duration of this culture period we noted differential decrease in levels of expression between the 3 groups: the unmodified (no SAR/CHS) and CHS receptors showed a substantial decrease in MFI up ranging from 22.4–49.5% compared with the day 3 MFI (Fig 4D compared with Fig 4C). This decrease in expression was expected and is likely due to responsiveness of the MoMLV LTR to T-cell activation state. After prolonged 7 day culture the MFI of vectors containing SAR was significantly higher than umodified. This decrease in expression was significantly less marked for the SAR-containing receptors (p< 0.02 for non codon optimized; see Fig 4D) suggesting that the SAR is functioning to prevent down-regulation of the GD2 CARs. The phenomenon of lower MFI at day 7 compared with Day 3 is observed equally in vectors containing or not containing the C to S mutation suggesting that non-specific activation of the suicide gene is not responsible for this silencing.

Following incubation with CID the levels of CAR expression of live PBMCs was assessed by flow cytometry (Fig 4B). As expected the C-to-S mutant receptors were refractory to CID treatment. Neither codon-optimization nor inclusion of the SAR sequence influenced CID response, but the CHS4 sequence appeared to result in greater levels of transduced T cells escaping CID treatment (Fig 4B). Escape from suicide gene activation appeared to be observed predominantly the low CAR-expressers and this was consistent with the lower overall expression observed for CHS4 receptors. Whilst a very small number of residual CAR-expressing T cells were detectable in the unmodified and SAR groups following CID treatment, it is unclear whether the cells retain sufficient CAR expression to display activity.

**Fig 5:**
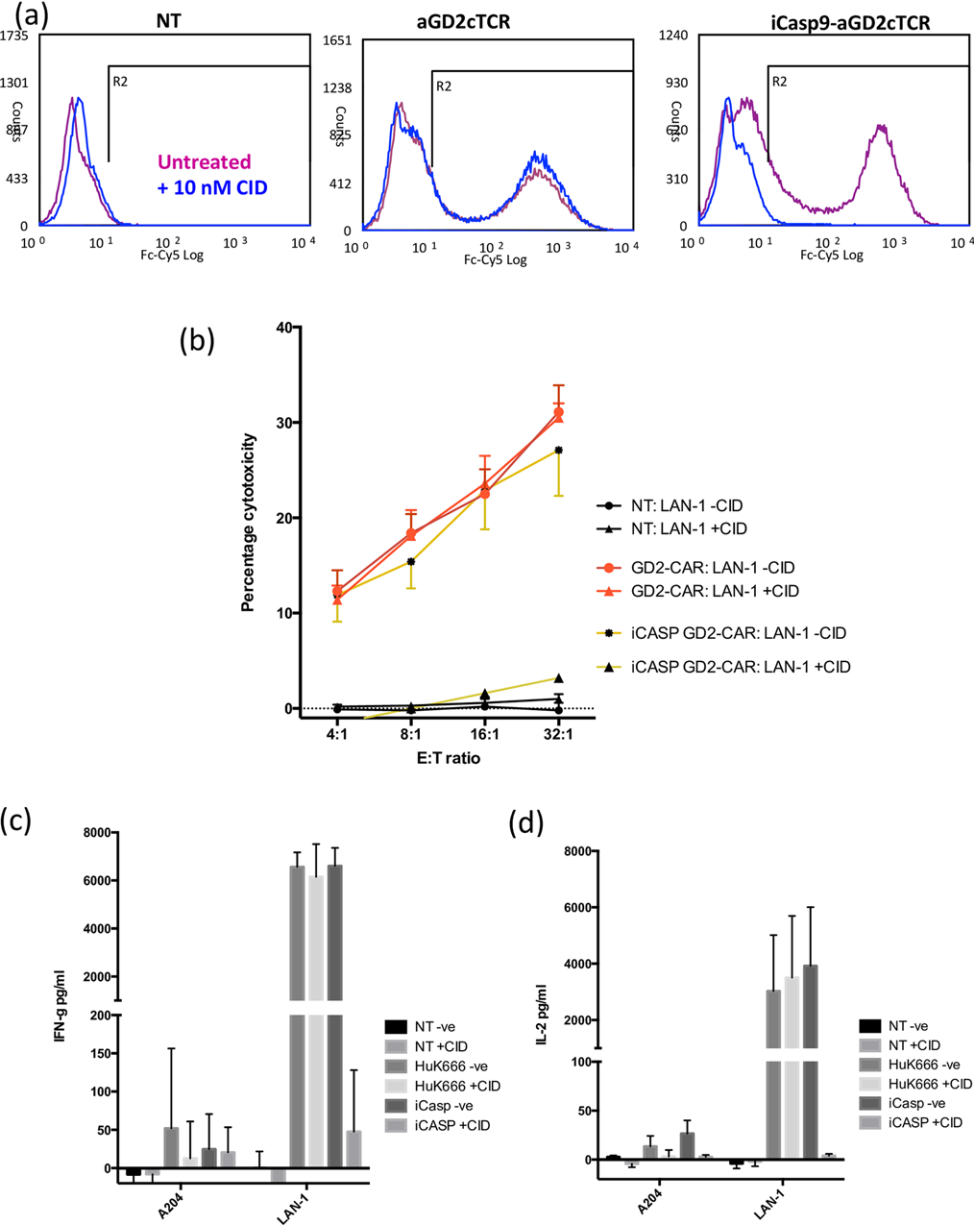
Consequence and function of iCasp9. The SAR codon-optimized cassette was taken further and compared with the original cassette without iCasp9. (a) Expression of the CAR was unchanged. Depletion is shown by facs after addition of CID. The function of cassettes with and without iCasp9 were assessed by (b) Killing (c) IFN-γ release and (d) IL-2 release. Data shown as means +/-SEM from 4 independent experiments with different donors.

Hence, in terms of expression, stability and response to CID, the codon-optimized SAR-containing receptor (iCasp-HuK) performed the best and this receptor was compared to the original HuK666 CAR for its capacity to mediate cytotoxicity and cytokine release in response to Lan1 cells (Fig 5). Transduced PBMCs were incubated for 72 hours in the absence or presence of 10nm CID and then co-cultured with Lan-1 or A204 cells. An example of the expression of these CARs in the presence and absence of CID is shown in Fig 5A. Levels of HuK666-iCasp9 expression were effectively eliminated by induction of the iCasp9 (Fig 5A).

HuK666-transduced PBMCs displayed comparable levels of cytotoxicity and cytokine release in response to Lan1 cells irrespective of prior culture with CID. In the absence of CID the HuK666-iCasp9 construct performed as well as the receptor lacking the suicide gene. However a 72-hr pre-treatment with CID abolished the release of cytokines by (Fig 5C and 5D) and the cytotoxicity of (Fig 5B) T cells expressing HuK-iCasp9.

### *In vivo* function of iCasp9-CAR cassette

**Fig 6:**
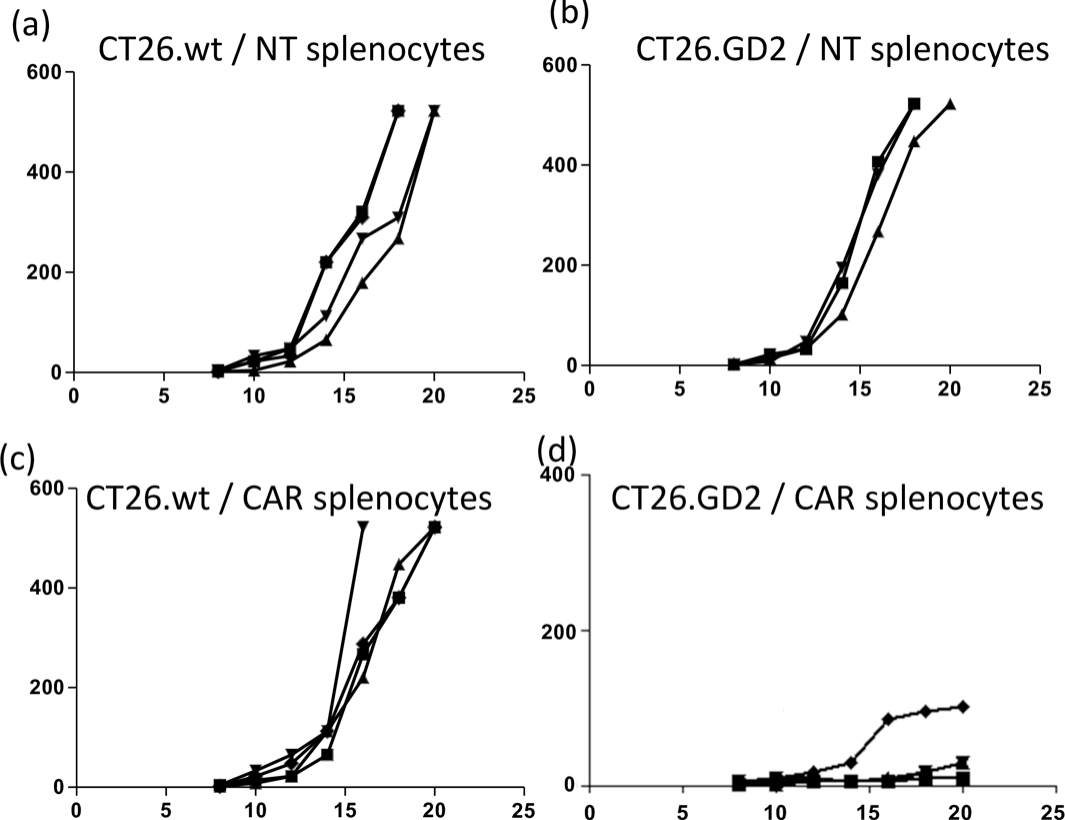
In vivo testing of GD2 CAR. Balb-C mice were innocultaed with 1x10^6^ CT26 or CT26-GD2 cell mixed in matrigel. 10 days post tumour inoculation, mice were sub-lethally irradiated (200 rads) and two weeks post tumour injection, mice were intravenously transplanted with a total of 1.5x10^6^ transduced splenocytes or non transduced controls by tail vein injection. Wild type CT26 were used as antigen negative controls (a,c) whereas CT26 –GD2 tumors treated with untransduced T cells (b) did not regress whilst CT26-GD2 tumors challenged with CAR T cells regressed. Each line represents one mouse.

To confirm that the efficacy and specificity of the co-expressed optimized CAR and suicide gene observed in vitro was likely to equate to clinical efficacy we evaluated the iCasp9-CAR cassette in an immunocompetent mouse model. The GD2 antigen is a ganglioside of identical chemical structure between species so the ScFv huK666 ScFv binds GD2 equally in mouse and human cells. We made use of the weakly immunogenic CT26 colon cancer cell line, which consistently forms tumours subcutaneously in Balb/c mice. CT26 cells were transduced with a gammaretrovirus encoding GD2 and GD3 synthases, and a clone (number 7) with bright GD2 expression was selected for analysis of GD2 targeting by the GD2-CAR in an immunocompetent model (S1 Fig). CT26-clone 7 cells induced specific release of IL-2 and IFN-γ following co-culture with splenocytes transduced with GD2-CAR (S2 Fig). In vivo, CT26-GD2 clone 7-derived tumors were efficiently eliminated by CAR transduced splenocytes in contrast to GD2 negative CT26 cells which grew at the same rate as tumors in mice treated with untransduced splenocytes (Fig 6).

## Discussion

High-risk Neuroblastoma represents an unsolved clinical problem. Neuroblastoma tumors express the diasialoganglioside GD2 abundantly and ubiquitously. GD2 is one of a few solid-tumor antigens with relatively little expression on other tissues, specifically GD2-low level expression on peripheral nerves, making GD2 an attractive target for CAR therapy. An early GD2 CAR T-cell study was performed which compared EBV-CTLs and peripheral blood T-cells transduced with CARs in the same patient. Responses were transient and only low-level persistence of CAR T-cells was observed [8].

A possible limitation of that study was use of a first generation [21] receptor, which only transmits immunological signal 1 upon ligation. Second-generation CARs have been described, which also transmit co-stimulatory signals [22]. A double marking comparison of first and second generation CARs suggests superiority of the latter [9]. Indeed an increasing body of clinical data with second generation CARs targeting CD19 CAR T-cell therapy in B-cell malignancies has confirmed the superiority of second generation CARs [23,24]. Here, we incorporated an endodomain which transmits two costimulatory signals, one each from the Ig superfamily coreceptor family (CD28) and the TNF family coreceptor family (OX40).

Increasing clinical experience has demonstrated that CARs incorporating binding domains derived from murine antibodies can elicit severe reactions in patients, which can contribute to enhanced clearance of transduced T cells and CAR activation with considerable toxicity [25,26]. This eventuality is more pressing in Neuroblastoma: since anti-GD2 mAb therapy is now standard-of-care, many patients will have been exposed to the chimeric 14–18 mAb. Notably these therapeutic antibodies are derived from the same clone as the CAR used by Pule et al. CARs containing 14.18-based scFv have been demonstrated recently to lead to tonic signalling sufficient to inhibit function [27]. Hence, we attempted to address the possibility of both pre-formed and CAR-induced anti-idiotype and anti-mouse framework response. We therefore used a different binder family, which was humanized.

The function of the receptor and the selection of the correct spacer may be important for clinical efficacy [13,14,21,28]. Guest et al have investigated how the sequence of the spacer and CAR function appears to relate to the distance of the receptor binding site on the target antigen from the target cell membrane [13]. The authors noted that in the case of NCAM and 5T4, antigens in which the CAR epitope is located adjacent to the membrane, their cognate CARs required the presence of an Fc spacer for optimal function. Removal of this spacer resulted in the decrease in cytotoxicity and IFN-γ secretion from transduced T cells in response to antigen-expressing tumour cells. The opposite appeared true in the case of CD19 and CEA, both antigens in which the CAR-binding site is further away from the membrane. CARs directed against these latter two antigens displayed reduced IFN-γ response if they contained the Fc spacer, although cytotoxicity was unaffected. Hudecek et al in the ROR-1 and CD19 models have suggested that optimal length and flexibility of spacer is also dependent on the location of a target epitope in relation to the cell surface [14,28].

We observed that alteration of the spacer in our GD2 CAR resulted in alterations in the cytotoxic potential and cytokine secretion of transduced PBMCs. Overall the Fc spacer provided the optimal response whilst both receptor variants containing the CD8 stalk appeared sub-optimal in terms of cytotoxicity and IL-2 release, although the stalk-variant-transduced PBMCs were consistently observed to secrete higher levels of IFN-γ upon stimulation than PBMCs transduced with the Fc variant. A lack of correlation between cytotoxicity, proliferation and cytokine secretion for certain receptor constructs has been noted previously [13] [29,30]. Transduction levels were consistently equal for all spacer variants and this did not appear to account for observed differences in function except in the case of the hinge variant, which was weakly expressed on the cell surface of transduced cells, and displayed little or no reactivity against tumour cells in any of the measured parameters. This may be due to limited access of the detecting anti-HA antibody to the HA tag on the hinge receptor or to a decreased stability on the cell surface although this receptor configuration has been observed to be functional in other systems[13,21]. Incorporating the hinge region into the stalk variant appeared negatively to influence receptor function and ameliorated cytokine secretion compared to the stalk alone. It has been postulated that the key feature of the spacer region to determine function is flexibility, allowing the binding domain to access the target antigen. One possibility is that inclusion of the hinge segment may decrease receptor flexibility and that this may overcome any benefit derived from a slight increase in receptor length.

Ultimately, we selected the Fc-spacer variant as being optimal in our hands and took this forward for further investigation. However one issue that has been noted with CARs containing the IgG Fc is the binding of this region to Fcγ receptors (FcγR) on cells of the innate immune system resulting in inappropriate off-target activation of the transduced T cells [16,17]. Mutation of key FcγR binding residues abrogates this interaction without affecting CAR function and such a modification may be necessary for effective anti-tumour responses in vivo whilst retaining the additional advantage of Fc as a spacer–including detection of CAR T cells with with polyclonal anti-Fc antibodies.

Gene modified, antigen-redirected T cells have an inherent potential to cause significant and long-lasting toxicity and such adverse events have been noted in clinical trials. An early trial of a 1^st^ generation CAR against carbonic anhydrase IX expressed on renal carcinoma resulted in on-target off-tumour hepatotoxicity due to the expression of this antigen on biliary epithelium [31]. Whilst this toxicity could be considered relatively mild, more severe and even fatal incidences have been subsequently documented. Rapid and fatal pulmonary toxicity was observed in a study utilizing HER2-CAR in the treatment of colon cancer patients, which was subsequently attributed to low level expression of the target antigen on the pulmonary epithelium [32] whilst 2 patients treated in a trial of a MAGE-A3 TCR developed fatal neurological toxicity due to the receptor recognizing an epitope which is shared amongst several MAGE family members [33] Experiences such as these demonstrate that the incorporation of an inducible suicide gene allowing the selective eradication of the transduced T cells, is an important feature of the CAR cassette. GD2 is expressed at low levels on some peripheral nerves and anti-GD2 antibody therapy can result in a transient pain syndrome; therefore in order to anticipate and prevent on-target, off-tumour toxicity directed against peripheral nerve GD2 we have incorporated the well-characterised inducible caspase 9 (iCasp9) suicide gene into our CAR cassette [11].

Linking the iCasp9 to the receptor using a viral 2A sequence ensures equal expression with the CAR and overcomes the reduction in downstream gene expression frequently seen with an IRES sequence. The additional transcriptional burden of co-expressing iCasp9 makes this more challenging. Achieving high and constant level of CAR expression on the transduced T cells may be key to achieving tumour rejection. Furthermore, transduced T cells would be required to sustain CAR expression over time even in a quiescent state. We have investigated ways to optimize our CAR cassette in order to achieve uniform, high levels of CAR expression by incorporating sequences known to have this effect on the transgene. Both the human β-interferon scaffold attachment region and chicken 5’HS4 beta globin chromatin insulator sequence have been shown to enhance long term retroviral transgene expression in human PBMCs and to prevent down-regulation of transgene expression as the cells become quiescent [19,20,34]. These properties seem to be due to the prevention of histone H3 deacetylation and CpG dinucleotide methylation in the 5’LTR region of the proviral integrant.

We observed that over 7 days post transduction PBMCS containing CAR constructs lacking either the CHS or the SAR experienced decreased expression of the CAR as judged by the mean fluorescence intensity of receptor staining by flow cytometry, possibly reflecting a reduction in the number of activated T cells in the culture over time. This effect was less pronounced in the presence of the SAR. We also noted that the SAR constructs consistently showed a higher expression 3 days post transduction and a much narrower distribution of receptor expression than the other constructs suggesting that the SAR appeared to be enhancing CAR expression by two different mechanisms. In contrast the CHS sequence did not appear to normalize expression and overall constructs containing this modification were poorly expressed. This is consistent with results in adipose tissue stem cells in which both enhancement sequences were capable of sustaining long term expression of a proviral transgene whilst the SAR alone was able to increase expression initially[20,35].

We found that the addition of iCasp9 to the optimized GD2-CAR-SAR vector did not reduce expression of the CAR when compared to a control construct containing a non-functional iCasp9 gene either initially or after 10 days in culture. Nor did the suicide gene have any effect on responses to Lan-1 cells in the absence of suicide gene activation. Activation of the suicide gene in transduced PBMCs through a 72 hour culture with CID reduced cytokine responses towards Lan-1 cells to levels comparable to those seen in the presence of GD2 negative A204 cells. Cytotoxicity was also severely ablated in CID treated cells although some activity was detectable at the highest effector:target ratios. Cloning a mini-operon for GD2 production allowed us to convert any cell line from GD2 negative to GD2 positive. This allowed us to test our CAR in a syngeneic mouse model using CT26 cells engineered to become GD2 positive. Our cassette was able to reject established tumour and administration of CID allowed depletion of CAR T-cells.

In summary, by modifying various elements of CAR design, incorporating an inducible suicide gene and DNA sequences which enhance, normalize and maintain transgene expression we have described here an optimized retroviral CAR vector to take forward to phase I clinical trial for neuroblastoma.

## Materials and Methods

### Molecular cloning

All constructs were generated by splicing by overlap PCR. Codon optimization was performed by de-novo gene-synthesis using PCR assembly of overlapping oligonucleotides (IDT DNA, Coralscille Ioha). Codon-opimization used an in-house algorithm (written by MP, available on request): the algorithm strove to keep GC content at 70% and eliminate cryptic splicing, harpins, literal repeats and any possible cis-acting sequences. Absolute identity of all constructs were sequenced by capillary sequencing (Beckman, UK). Phusion polymerase, quick Ligase and NEB5alpha (New England Biolabs, New England) were used for molecular cloning. Oligonucleotides were purchased from IDTDNA (Coralsville, Iowa). The retroviral vector used in all constructs was the splicing oncoretroviral vector SFG [36]. A truncated CD34 marked gene was co-expressed using fin-frame foot-and-mouth-like 2A peptide TaV. In later constructs iCasp9 was also co-expressed with CAR by cloning it upstream of the CAR separated by an in-frame 2A peptide[37]. Template for the chicken beta-globin insulator was a generous gift from Donald Kohn^14^ Template for SAR plasmid generous gift from Donald Kohn [20]

### Retroviral production and transduction

RD114 pseudotyped supernatant to transduce human T-cells was generated as follows: 293T cells were transfected with vector plasmid; RDF, an expression plasmid supplying RD114 envelope (gift of Mary Collins, University College London) [38] and PeqPam-env, an expression plasmid supplying gagpol (gift of Elio Vanin, Baylor College of Medicine)<9642098, 1658374>. Transfection was facilitated using Genejuice (Calbiochem, California). Eco pseudotyped supernatant to transduce murine splenocytes was generated in a similar way except RDF was substituted for by pMono.Eco, an expression plasmid supplying ecotropic envelope (gift of Gianpietro Dotti, University of North Carolina). Supernatant was harvested at 48 hours, refrigerated and pooled with 72 hour harvest. Transduction of human T cells was performed as follows: T-cells were isolated by Ficoll gradient centrifugation and stimulated with PHA at 5μg/ml OKT3 / CD28 (Miltenyi clone 15E8) monoclonal antibodies at 1μg/ml. IL-2 was added at day one. On day 3, T-cells were harvested, plated on retronectin and retroviral supernatant and centrifuged at 1000xG for 40 minutes. Murine splenocytes from 6 to 8 week old female Balb/c mice were stimulated with Concavalin A / IL-7 (2 μg/mL and 1ng/mL respectively), then harvested, resuspended in fresh media and transduced in untreated 24-well plate pre-coated with 0.5 ml of retronectin solution (20μg/ml). To preload the vector, the retronectin solution was removed, and 1 ml of the vector was added. The plate was centrifuged at 1000g for 40 minutes at RT. Following transduction, splenocytes were re-stimulated with IL-2 (50ng/mL) and cultured overnight.

### Cell Lines and Primary T-cell culture

Lan-1 cells, A204 and SupT1s were purchased from the ECACC. Lan-1 and A204 cells were cultivated in IMDM supplemented with 10% FCS and L-Glutamine. CT26 cells were purchased from ATCC and were transduced with a gammaretroviral cassette co-expressing GD2 and GD3 synthases to drive the biosynthesis of GD2. A single cell clone (clone 7) with stable and high GD2 expression was selected for *in vivo* studies.

### Antibodies and Flow Cytometry

Expression of exogenous HuK and MuK CAR constructs was monitored by flow cytometry using a Cy5-conjugated polyclonal goat anti-human Fc antibody (Jackson Immuno). Expression of HuK spacer variants was assessed by co-staining with CD34-APC (BD) and anti-HA-PE (Santa Cruz Biotech) antibodies. Cells were routinely stained with antibodies for 30 minutes on ice and washed once with PBS. Flow cytometric analysis of stained cells was performed using a Beckman Coulter Cyan ADP and data were analysed in Summit Flow Cytometry software Ver 4.3.

### Cytotoxicity assays

CAR-mediated cytotoxicity was measured in a 4-hour 51Cr-release assay utilizing either GD2-bearing Lan-1 neuroblastoma cells, GD2-negative A204 rhabdomyosarcoma cells, the human monocyte cell line THP-1, or SupT1 cells as targets as indicated. PBMCs were transduced as described with equal titres of the indicated CAR retroviral supernatant. Levels of CAR expression were assessed by flow cytometry. Prior to the assay, transduced cultures were depleted of CD56-expressing cells using CD56-microbeads (Miltenyi Biotech) according to the manufacturer’s instructions. Target cells were loaded with 3.7 MBq 51Cr per 10^6^ cells for 1 hour at 37C and washed 5 times in culture medium to remove unincorporated chromium. Assays were carried out in triplicate at effector:target ratios of 32:1, 16:1, 8:1 and 4:1 in 96-well V-bottom plates. T cells transduced with an anti-CD19 CAR were included as a further control for non-specific CAR-meditated cytotoxicity. Supernatants were harvested and 51Cr levels measured by gamma counting using a 1282 Compugamma Gamma Counter (LKB Wallac). Readings for background or spontaneous release were determined by incubating targets alone, and maximum release was estimated by incubating targets with 0.1% Triton X. Killing was defined as [(Experimental release–background release x100]/(Maximum release–background release).

### Proliferation assays and Cytokine release

In order to measure CAR-mediated T cell proliferation and cytokine release 1x10^6^ transduced PBMCs were co-cultured with an equal number of GD2-positive or GD2-negative target cells. After incubation for 72 hours at 37C aliquots were taken and T cell numbers assessed by trypan blue exclusion. Supernatants from these co-cultures were harvested and stored at -20C for subsequent cytokine analysis. Cells were resuspended in fresh culture medium, incubated at 37C for a further 96 hours and subsequently counted. IL-2 and IFN-γ levels in harvested co-culture supernatants were measured using Human Duoset Elisa kits (R and D systems) according to the manufacturer’s instruction.

### Induction of cell killing through iCasp9

1x10^6^ PBMCs transduced as described above with the indicated iCasp-GD2-CAR construct were incubated for 7 days at 37°C in the presence of 100u/ml IL-2 and 10nm chemical inducer of dimerization (CID) or an equal volume of ethanol, the vehicle for the CID. Live cells were identified by annexin V/PI staining and the expression of GD2 CAR on live cells was assessed by flow cytometry.

### In vivo tumor growth assays

Animal experiments were performed in accordance with UK Home Office license authorization and in accordance with the ARRIVE guidelines. Mandated monitoring includes daily inspection of all experimental animal for health and wellbeing. Mandated humane endpoints include a maximal tumor diameter of 15mm and euthanizing of animals prior to maximal tumor dimention or on first identification of ill health (ruffled fur or decreased activity) whichever occurs first. No mice died prior to euthanasia. Groups of mice (BALB/c mice, female aged 6 to 8 weeks) were inoculated subcutaneously with 1x10^6^ CT26 or CT26-GD2 cell mixed in matrigel. 10 days post tumour inoculation, mice were sub-lethally irradiated (200 rads) and two weeks post tumour injection, mice were randomized for intravenous transplantation with a total of 1.5x10^6^ transduced splenocytes or non transduced controls by tail vein injection. Treated mice underwent *in vivo* monitoring of tumor growth using electronic calipers, Mice were sacrificed using schedule 1 methods when the tumour volume exceeded 1.5 cm^3^ or when they were otherwise unwell.

### Statistical analysis

Data was analysed in Prism. Significance of differences between experimental groups was determined using the Mann-Whitney U test.

## Supporting Information

S1 Fig Transgenic expression of GD2 in murine cell line CT26. Top: Murine CT26 colon carcinoma cells were transduced with SFG gammaretroviral vector driving expression of GD2 and GD3 synthases separated by self cleaving 2A sequence. Bottom; GD2 expression measured by flow cytometry in CT26-GD2 clone 7, selected for in vivo experiments.
(TIFF)

S2 Fig Function of murine splenocytes against GD2 expressing targets. IL-2 (a) and Interferon gamma (b) secretion following culturing of GD2-CAR transduced splenocytes with wild type CT26 or GD2 positive CT26-clone 7.
(TIFF)
