# Supplementary figures and images for: An Optimized GD2-Targeting Retroviral Cassette for More Potent and Safer Cellular Therapy of Neuroblastoma and Other Cancers
Source: PLoS One. 2016 Mar 31;11(3):e0152196. doi: 10.1371/journal.pone.0152196 (PMC4816271; doi:10.1371/journal.pone.0152196)

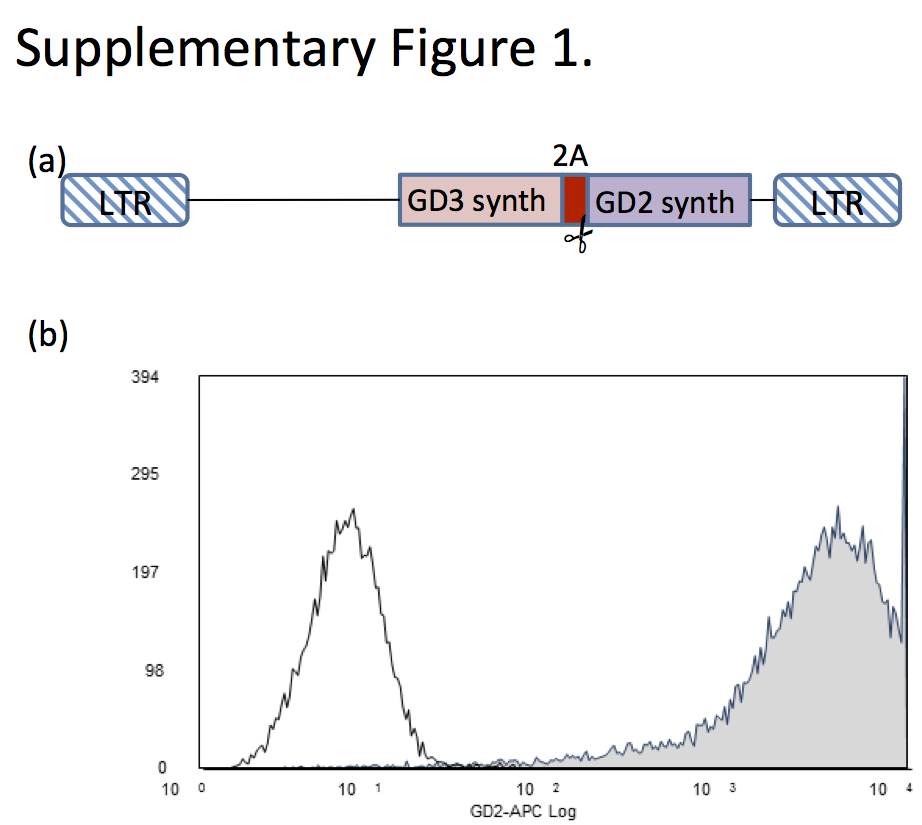

Supplement: S1 Fig — Top: Murine CT26 colon carcinoma cells were transduced with SFG gammaretroviral vector driving expression of GD2 and GD3 synthases separated by self cleaving 2A sequence. Bottom; GD2 expression measured by flow cytometry in CT26-GD2 clone 7, selected for in vivo experiments. (TIFF) [file pone.0152196.s001.tiff]

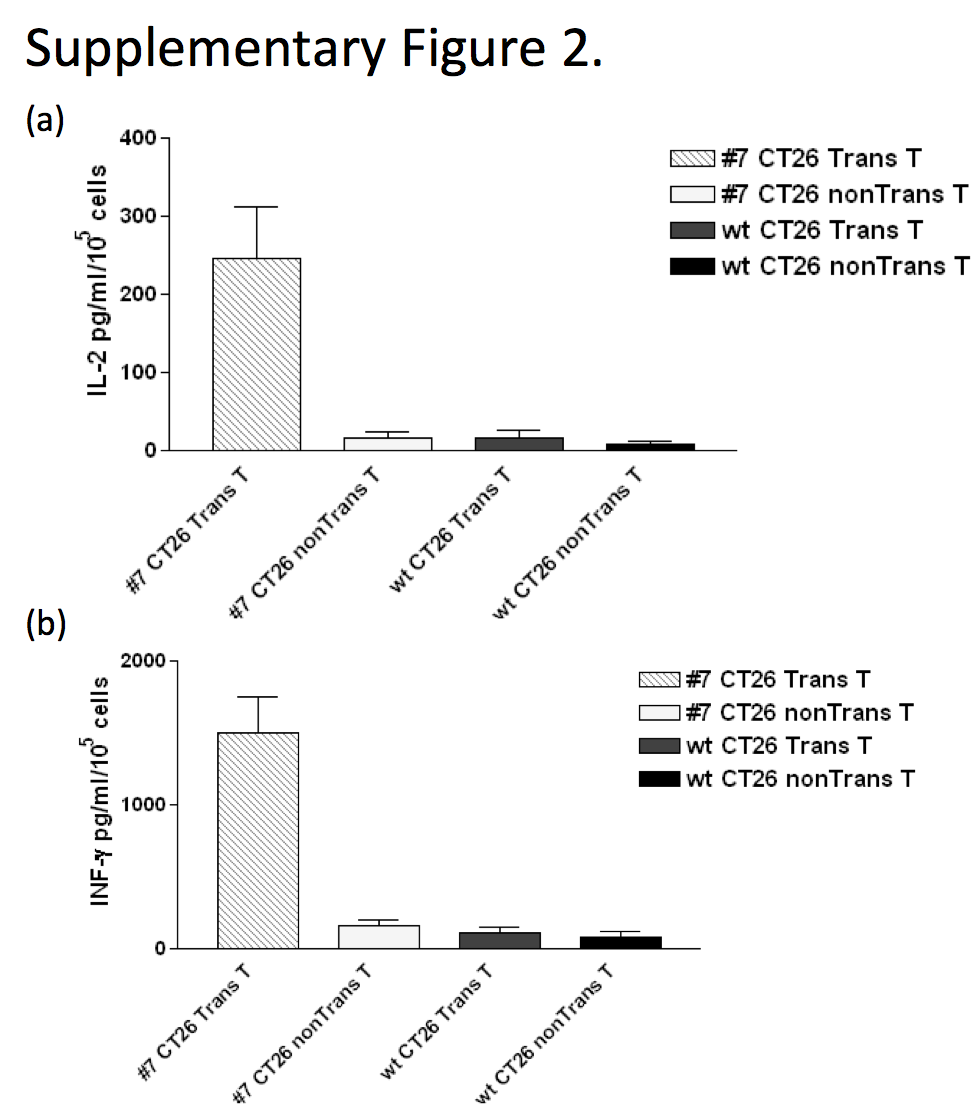

Supplement: S2 Fig — IL-2 (a) and Interferon gamma (b) secretion following culturing of GD2-CAR transduced splenocytes with wild type CT26 or GD2 positive CT26-clone 7. (TIFF) [file pone.0152196.s002.tiff]
